# National Black HIV/AIDS Awareness Day — February 7, 2019

**DOI:** 10.15585/mmwr.mm6804a1

**Published:** 2019-02-01

National Black HIV/AIDS Awareness Day is observed each year on February 7 to highlight the continuing disproportionate impact of human immunodeficiency virus (HIV) infection and acquired immunodeficiency syndrome (AIDS) on the U.S. black/African American (black) population.

In 2017, blacks represented 13% of the U.S. population (*1*), but accounted for 44% of all new HIV diagnoses (*2*). Among racial/ethnic groups, the highest rate of new HIV diagnoses occurred among blacks (41.1 per 100,000 population). Blacks also had the highest rate of new diagnoses of HIV infection in each of the four census regions of the United States; the highest overall rate was among blacks in the South (44.8 per 100,000 population).

Partner services is an effective, high-yield strategy for identifying undiagnosed HIV infections and thereby linking persons with newly diagnosed HIV infection into HIV care. A study reported in this issue of *MMWR* presents the first national level analysis of HIV partner services offered to blacks through CDC-funded health departments (*3*). CDC supports a range of efforts to reduce the risk for acquiring or transmitting HIV infection among blacks (https://www.cdc.gov/features/BlackHIVAIDSAwareness).
